# Calculation of and Key Influencing Factors Analysis on Equivalent Resilient Modulus of a Submerged Subgrade

**DOI:** 10.3390/ma17040949

**Published:** 2024-02-18

**Authors:** Junyao Tang, Siyu Chen, Tao Ma, Binshuang Zheng, Xiaoming Huang

**Affiliations:** 1 School of Transportation, Southeast University, Nanjing 211189, China; tjyseu@163.com (J.T.); chsy@seu.edu.cn (S.C.); 2 School of Modern Posts, Nanjing University of Posts and Telecommunications, Nanjing 210023, China; zhengbs@njupt.edu.cn; 3 National Demonstration Center for Experimental Education of Road and Traffic Engineering, Southeast University, Nanjing 211189, China

**Keywords:** submerged subgrade, equivalent resilient modulus, constitutive model, finite element method, fluid–solid coupling

## Abstract

To calculate and analyze the equivalent resilient modulus of a submerged subgrade, a constitutive model considering the effect of saturation and matrix suction was introduced using ABAQUS’s user-defined material (UMAT)subroutine. The pavement response under falling weight deflectometer (FWD) load was simulated at various water levels based on the derived distribution of the resilient modulus within the subgrade. The equivalent resilient modulus of the subgrade was then calculated using the equivalent iteration and weighted average methods. Based on this, the influence of the material and structural parameters of the subgrade was analyzed. The results indicate that the effect of water level rise on the tensile strain at the bottom of the asphalt layer and the compressive strain at the top of the subgrade is obvious, and its trend is similar to an exponential change. The equivalent resilient modulus of the subgrade basically decreases linearly with the rise in the water level, and there is high consistency between the equivalent iteration and weighted average methods. The saturated permeability coefficient and subgrade height have the most significant effect on the resilient modulus of the subgrade, which should be emphasized in the design of submerged subgrades, and the suggested values of the resilient modulus of the subgrade should be proposed according to the relevant construction conditions.

## 1. Introduction

Abundant rainfall in tropical regions like Africa and Southeast Asia causes the seasonal submersion of the subgrade. Variations in moisture greatly affect the subgrade’s resilient modulus, which, in turn, reduces soil stiffness and increases structural reactivity under pavement loads [1,2,3]. A large number of studies have been carried out to discuss the effects of moisture content [4,5], matrix suction [6,7], loading frequency [8,9], confining stress [10], and soil type [11,12,13] on the resilient modulus. In practice, it is difficult to take into account the influence of so many factors at the same time. The most important ones are stress state and moisture state [14]. Through a large number of laboratory dynamic resilient modulus tests, the resilient modulus prediction equation was established to quantitatively describe the relationship between the resilient modulus and physical property parameters, state variables and environmental variables [15,16,17]. The mechanistic-empirical pavement design guide (MEPDG) design method incorporates the resilient modulus prediction equation as an intrinsic model of the subgrade and the granular layer to carry out the structural nonlinear analysis of pavement [18,19,20,21], as shown in Equation (1):

(1) $$M_{r}=k_{1}p_{a}{\left(\frac{\theta }{p_{a}}\right)}^{k_{2}}{\left(\frac{\tau_{oct}}{p_{a}}+1\right)}^{k_{3}}$$

where *M_r_* is the resilient modulus, MPa; *θ* is the bulk stress, kPa; *τ_oct_* is the octahedral shear stress, kPa; *p_a_* is the atmospheric pressure, usually taken as 100 kPa; and *k*_1_, *k*_2_, and *k*_3_ are material parameters.

On this basis, state variables characterizing moisture are added to the equation, and equations that consider both the stress and moisture states of the material were developed [22]. Liang et al. [23] proposed a moisture-dependent prediction equation based on the Bishop effective stress. Similarly, Qian et al. [24] replaced the parameter reflecting the contribution of matrix suction with a saturation-related parameter. Gu et al. [25], on the other hand, introduced volumetric moisture content to capture the effect of humidity, while adding an adjustable saturation factor for error reduction. Furthermore, Zhang et al. [26] proposed a new resilient modulus model to incorporate relative compaction in addition to matrix suction and the stress state. Liu et al. [27] collected and analyzed the existing resilient modulus models of subgrade unsaturated soils, and summarized the characteristics and scope of application of each model.

Due to the stress and moisture dependence of the resilience modulus, its spatial distribution is inhomogeneous [28,29]. Determining the equivalent resilient modulus entails replacing a series of resilient modulus values with a representative value based on the equivalence principle. In the design of pavement structures, the commonly chosen equivalence metric is the mechanical response or the service life of the structure in question [30]. Among the available mechanical response metrics, the most widely used one is deflection [31]. The equivalent resilient modulus is determined using the iterative inverse calculation method according to the principle of deflection equivalence [32,33]. In AASHTO, the current service index of pavement is used as an index to determine the empirical equation for the relative loss coefficient for different subgrade moduli, and the effective resilient modulus of the subgrade is back-calculated based on the average relative loss coefficient [34]. By referring to this idea, researchers predicted the attenuation of the resilient modulus of a subgrade using easily measured indicators, such as the compressive strain at the top of the subgrade, the number of load repetitions, etc., and established corresponding empirical models [35,36]. These empirical methods facilitate the determination of the modulus, but their accuracy depends on a large number of field tests and is highly dependent on the region in question.

In summary, although many moisture-dependent soil resilient modulus constitutive models have been proposed and verified using triaxial tests, most of them stay in the theoretical research stage, and there are few studies incorporating the finite element method to study the distribution of the modulus field and the structural mechanical response of the subgrade structure. Meanwhile, the methods for calculating the equivalent resilient modulus of subgrade are mostly empirical, and the process is simple and lacks systematic theoretical guidance; thus, whether it can be directly used for the resilient modulus calculation of a submerged subgrade needs to be further discussed. Consequently, analyses on the equivalent resilient modulus of a subgrade under different submerged conditions and subgrade parameters are less frequent, making it difficult to provide effective guidance for submerged subgrade design. Therefore, it is necessary to propose a procedural approach to simplify the calculation and analysis of the equivalent resilient modulus of a subgrade.

Accordingly, in order to efficiently calculate the mechanical response and equivalent resilient modulus of a submerged subgrade and analyze the key influencing factors of the resilient modulus, a suction-dependent resilient modulus constitutive model was applied to reflect the effect of the moisture field on the mechanical properties of a subgrade based on ABAQUS’s user-defined material (UMAT) subroutine. Subsequently, the pavement response was analyzed, and the equivalent resilient modulus was calculated through the equivalent iteration and weighted average methods via ABAQUS python script. Furthermore, the influence of key factors on the equivalent resilient modulus was analyzed, including material parameters and the structural parameters of the subgrade. This study provides a complete framework for the rapid calculation of the subgrade equivalent resilient modulus, and the analyzed results can be utilized as a guide for subgrade design and water damage mitigation.

## 2. Materials and Methods

### 2.1. Typical Submerged Subgrade Model

**Figure 1:**
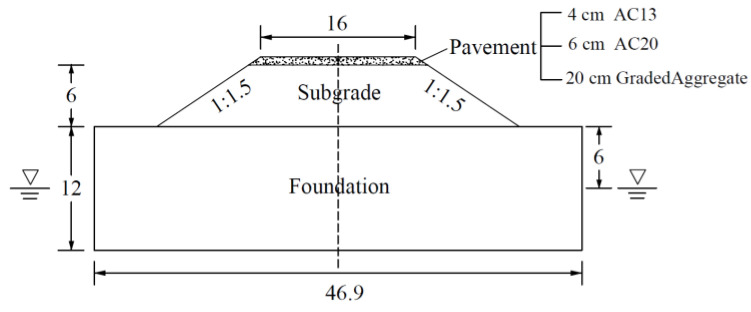
Typical highway structure (unit: m).

The typical highway structure applied in this study is shown in Figure 1, together with information on highway construction and hydrogeology in Africa. The pavement is composed of 4 cm AC13 + 6 cm AC20 + 20 cm graded aggregate with a width of 16 m. The slope of the subgrade was set to 1:1.5, with a height of 6 m. The foundation was assumed to be 12 m deep, with the initial water level being 6 m underground.

The parameters of the pavement material are shown in Table 1. We used dynamic moduli for all the elastic moduli *E* in order to replicate the structural performance under the field conditions.

The parameters of the subgrade and the foundation were set as follows: elastic modulus *E* = 35 MPa, Poisson’s ratio *ν* = 0.3, cohesion *c* = 15 kPa, and friction angle *φ* = 30 °. The relationship between permeability coefficient *K_w_* and saturation *S_r_* in the unsaturated zone is shown in Equation (2):

(2) $$K_{w}={S_{r}}^{3}K_{ws}$$

where *K_ws_* is saturated permeability coefficient, taken as 5 × 10^−7^ m·s^−1^.

To account for the seepage problems in unsaturated soil, the SWCC (Soil–Water Characteristic Curve) was defined using the Fredlund and Xing model [37]:

(3) $$\theta_{w}=C\left(\psi \right)\frac{\theta_{s}}{{\left\{ln\left[e+{\left(\frac{\psi }{a}\right)}^{n}\right]\right\}}^{m}}$$

(4) $$C\left(\psi \right)=1-\frac{ln\left(1+\frac{\psi }{\psi_{r}}\right)}{ln\left(1+\frac{10^{6}}{\psi_{r}}\right)}$$

where *θ_w_* is volumetric moisture content; *θ_s_* is saturated volumetric water content; *C*(*ψ*) is correction function; *ψ* is matrix suction, given in kPa; *ψ_r_* is residual matrix suction, given in kPa; and *a*, *n*, and *m* are fitting parameters, taken as 2813, 0.4836, and 3.7106, respectively.

**Figure 2:**
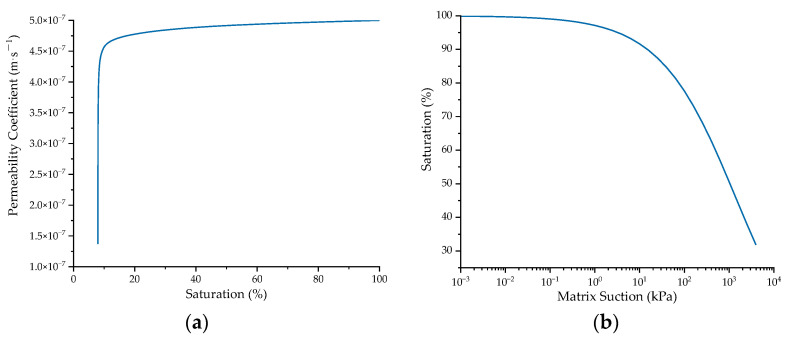
Hydraulic parameter curves of soil material: (**a**) relationship between permeability coefficient and saturation; (**b**) SWCC (Soil–Water Characteristic Curve).

The corresponding hydraulic parameter curves developed according to Equations (2)–(4) are shown in Figure 2.

**Figure 3:**
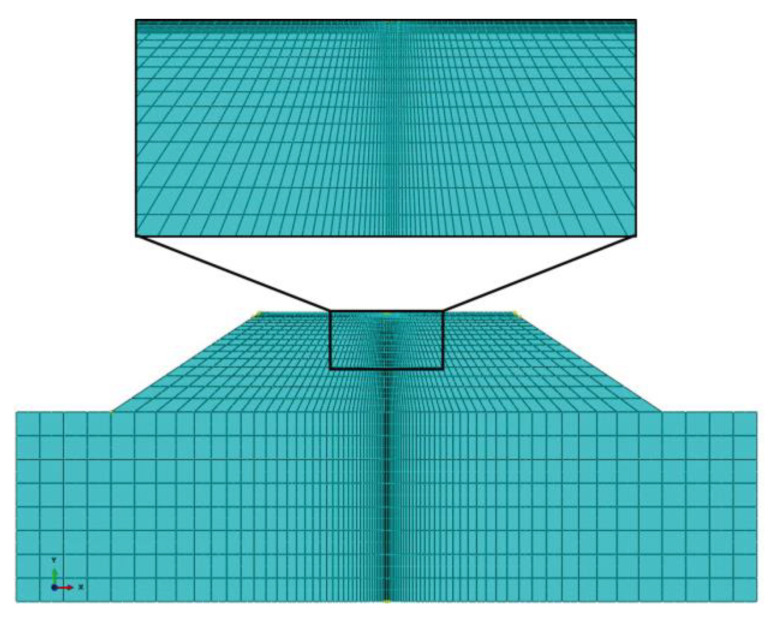
Mesh partition and local encryption.

The sides of the slope and foundation were set as the submerged boundary, and the boundary conditions related to pore water pressure were incorporated. In order to effectively carry out the fluid–solid coupling analysis, the plane pore water pressure units, CPE8P, were adopted in the subgrade and foundation. Since falling weight deflectometer (FWD) loads need to be applied subsequently to observe the mechanical response of the subgrade and pavement and back-calculate the resilient modulus of the subgrade accordingly, in order to improve the accuracy of the results, the mesh was divided into a local refinement set in addition to the global distribution. The middle part of the mesh was gradually encrypted from the outside to the inside, and the meshes of the surface layer and the base layer were encrypted vertically. The corresponding results are shown in Figure 3.

### 2.2. Constitutive Model of Resilent Modulus

**Figure 4:**
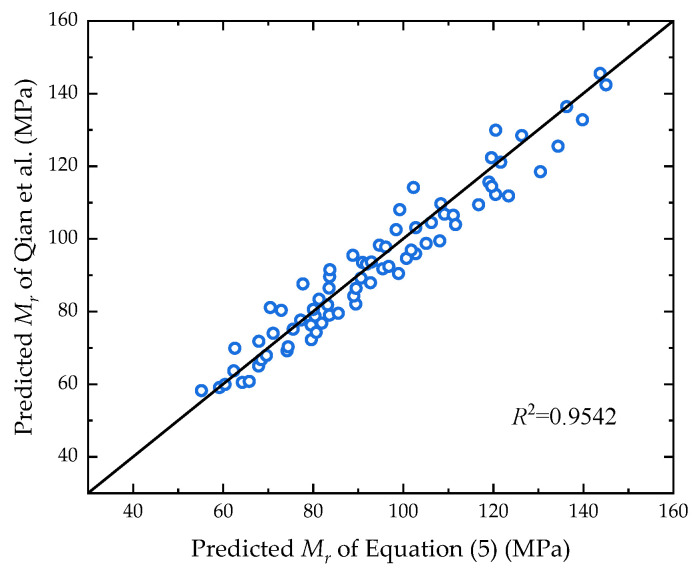
Correlation analysis of two prediction models [24].

Since a constitutive model of the unsaturated soil resilient modulus is not provided in ABAQUS, it was necessary to complete the definition with the assistance of the UMAT subroutine. Given the stress dependence and moisture dependence of the resilient modulus in unsaturated soil, a constitutive model considering the effect of saturation and matrix suction was applied based on Equation (1), as shown in Equation (5):

(5) $$M_{r}=k_{1}p_{a}{\left(\frac{\theta +3S_{r}\psi }{p_{a}}\right)}^{k_{2}}{\left(\frac{\tau_{oct}}{p_{a}}+1\right)}^{k_{3}}$$

where the fitting parameters *k*_1_, *k*_2_, and *k*_3_ were determined using nonlinear regression analysis, and the source data came from the predicted resilient modulus of unsaturated clay in Nantong, Jiangsu Province, from Qian’s research [24]. As shown in Figure 4, the horizontal and vertical coordinates of the dots represent the results of the resilient modulus predicted using the two different models respectively. The fitting parameters were obtained as *k*_1_ = 0.4351, *k*_2_ = 0.9698, and *k*_3_ = −1.6522, and the goodness-of-fit value of the two models was 0.9542, with a significant correlation.

Under the submerged condition, the stress state at each node of the subgrade was extracted by the UMAT subroutine through the interface with ABAQUS, and the corresponding resilient modulus was calculated based on the constitutive model. Subsequently, the stress state was updated according to the new distribution of the resilient modulus. When the water level rises, the states of stress and moisture change correspondingly, leading to differences in the resilient modulus and the pavement response.

### 2.3. Calculation of Equivalent Resilient Modulus

Due to the stress and moisture dependence of the resilient modulus in unsaturated soil, the stress and moisture states in the subgrade vary from position to position, resulting in an inhomogeneous spatial distribution. The equivalent resilient modulus is a representative value of the spatial distribution according to a given equivalence principle used to reflect the overall bearing capacity of the subgrade. For this purpose, the equivalent resilient modulus of the subgrade was calculated using the equivalent iteration and weighted average methods.

#### 2.3.1. Equivalent Iteration

For the design of flexible base asphalt pavement, the tensile strain at the bottom of the asphalt layer, *ε_t_*, and the compressive strain at the top of the subgrade, *ε_c_*, are regarded as key indicators in structural calculations, controlling fatigue damage and permanent deformation, respectively, and both can be adopted as equivalent indicators for iteration [38,39].

**Figure 5:**
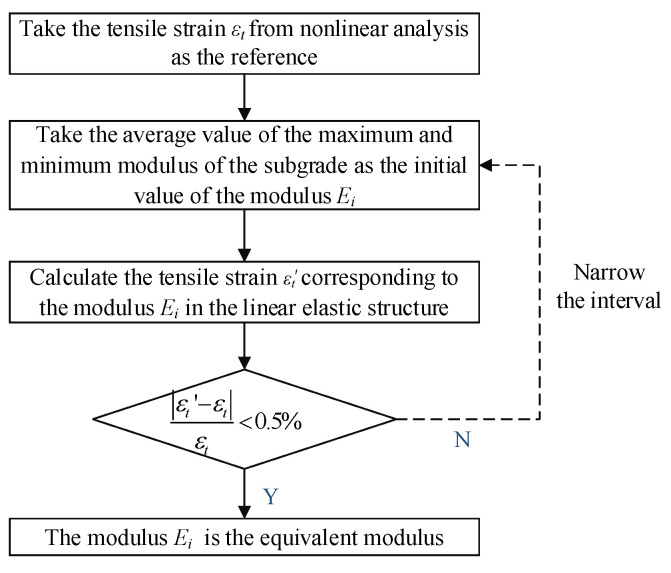
Equivalent iteration process of subgrade equivalent resilient modulus.

The iteration process based on *ε_t_* is shown in Figure 5. The iteration algorithm was implemented via a python script. The steps are as follows:

1. Set the reference value. The value *ε_t_* was calculated using the nonlinear model under the peak load.
2. Calculate the linear elastic response. A linear elastic model was established with the initial modulus *E_i_* between the maximum and minimum values in the modulus distribution of the nonlinear model, and the corresponding response *ε_t_’* under the same load was calculated.
3. Iterative convergence algorithm. The convergence criterion is that the error between the linear elastic response and the reference value must be less than the permitted value (0.5%), and the bisection method was applied to ensure convergence of the iterations.

#### 2.3.2. Weighted Average

The resilient modulus distribution of the subgrade was calculated according to Equation (5). On this basis, determining the weighted average involved finding a suitable weight function to calculate the equivalent resilient modulus of the subgrade directly.

**Figure 6:**
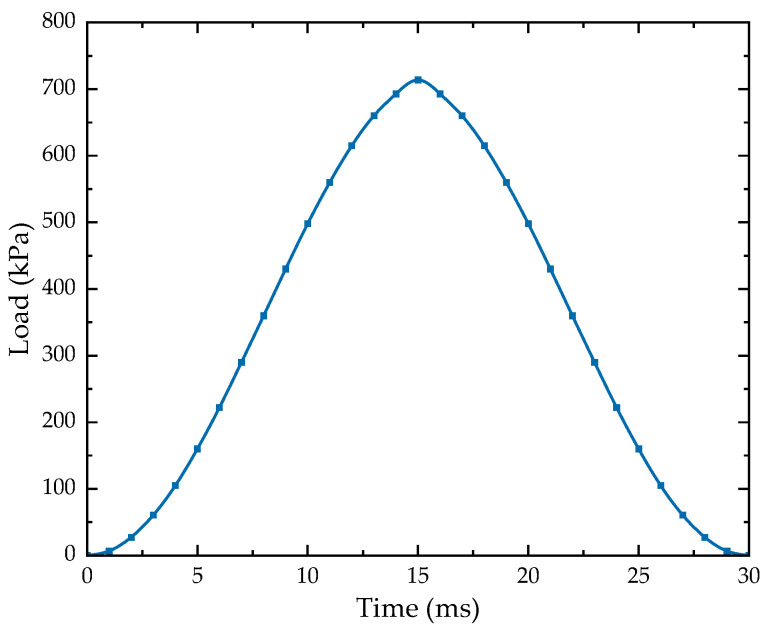
FWD load time-history curve.

Generally speaking, the closer the soil is to the center of the load, the higher the load it is subjected to, and, accordingly, the greater its contribution to the equivalent resilient modulus. Thus, the weight function should reflect this characteristic. To this end, a falling weight deflectometer load was applied to the model to analyze the dynamic response. The FWD load was simplified as a circular homogeneous load with a half-period sinusoidal function with a peak load of 0.714 MPa, an action time of 30 ms, and a loading radius of 0.15 m, as shown in Figure 6. Considering the two-dimensional plane model used in this study, the circular homogeneous load was converted to a line load with a peak load of 168.23 kPa.

**Figure 7:**
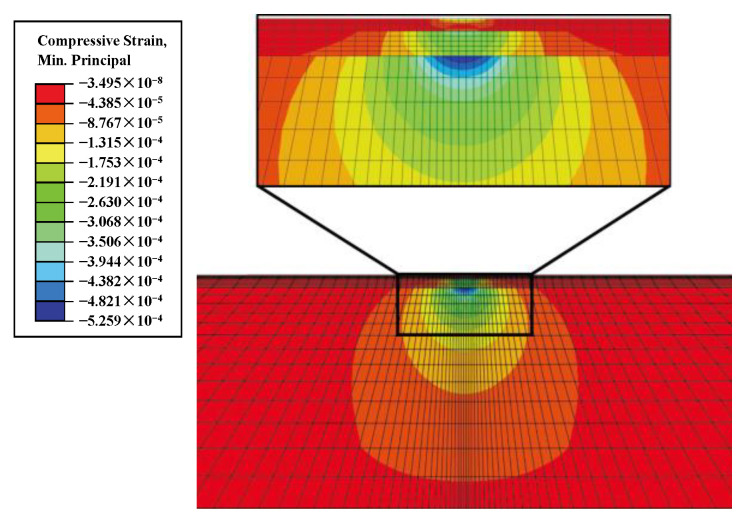
Distribution of maximum compressive strain at FWD peak load. (Unit: με).

The result of the top compressive strain of the subgrade *ε_c_* at the peak load is shown in Figure 7, from which it can be seen that the spatial distribution of the compressive strain meets the requirements for the weight function, which can be used to calculate the weighted average of the equivalent resilient modulus according to Equation (6):

(6) $$E=\frac{\sum E_{i}\cdot \varepsilon_{ci}}{\sum \varepsilon_{ci}}$$

where *E* is the equivalent resilient modulus; *E_i_* and *ε_ci_* are the resilient modulus and maximum compressive strain at the center of the elements within the subgrade.

## 3. Results

### 3.1. Validation of Constitutive Model

In order to verify the validity of the proposed constitutive model, as shown in Equation (5), the results of laboratory tests reported in the study by Qian et al. [24] and Liang et al. [23], which include clay samples from different regions of China and the USA, were selected for analysis, and the results of the resilient modulus of the soil samples under different matrix suction conditions were obtained through triaxial tests.

**Figure 8:**
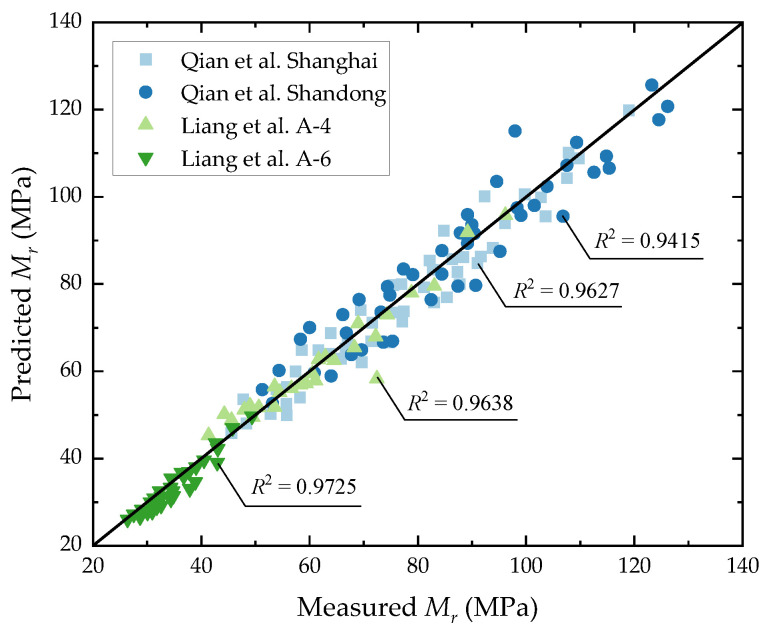
Comparison of predicted and measured resilient moduli for different soil samples in Qian et al. [24] and Liang et al. [23].

The model of Equation (5) was used to fit the above test data nonlinearly, and the obtained fitting parameters are summarized in Table 2. The fitting results are shown in Figure 8, wherein the goodness of fit is above 0.94. It can be seen that the results predicted by the model have a high degree of fitting with respect to the measured results of the laboratory tests, making this model highly applicable to different soil samples of subgrades in different regions.

### 3.2. Distribution of Resilient Modulus

In order to illustrate the effect of water level variations on the distribution of field variable outputs, the final states with the initial water level and the water level raised to 2 m were chosen for analysis.

**Figure 9:**
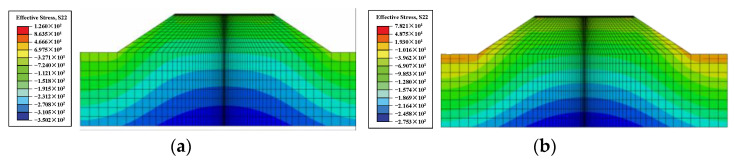
Distribution of vertical effective stress (**a**) at a water level of 6 m underground and (**b**) at a water level of 2 m (Unit: kPa).

The vertical effective stress distribution of the initial state obtained from the analysis is shown in Figure 9a. Due to the existence of side slopes, the vertical effective stress shows a gradual increase from the surface of the side slopes inwards. As the water level rises and reaches stability, the vertical effective stresses in all parts of the structure are reduced significantly, as shown in Figure 9b, which is consistent with the results of the theoretical analysis.

**Figure 10:**
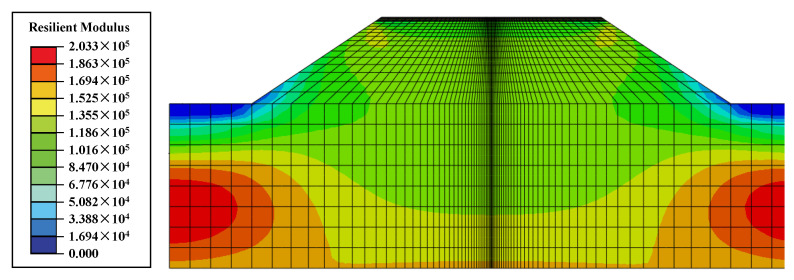
Distribution of resilient moduli at a water level of 2 m (Unit: kPa).

The distribution of the resilient modulus at a raised water level of 2 m is shown in Figure 10. In general, the resilient modulus gradually increases from the top of the subgrade and the slope to the depth of the foundation, which is consistent with the stress field distribution in Figure 7. From Equation (5), it can be gleaned that the contributions of bulk stress and shear stress to the resilient modulus are opposite, which means that the value of bulk stress is relatively large compared with that of shear stress and plays a dominant role in the magnitude of the resilient modulus. Meanwhile, the effect of matrix suction is reflected in the stress results through Bishop’s effective stress principle, and the foot of the slope has the smallest resilient modulus due to the small bulk stress and the presence of pore water pressure. At the top of the subgrade, the resilient modulus is larger than that at the foot of the slope; this is because the presence of matrix suction makes the effective stress here significantly higher.

### 3.3. Dynamic Response

In order to analyze the dynamic response characteristics of the submerged subgrade, the variations in the tensile strain at the bottom of the asphalt layer *ε_t_* and the compressive strain at the top of the subgrade *ε_c_* at different water levels were analyzed. The strain results discussed in the following are the maximum principal strains of the elements at the FWD peak load in Figure 6.

**Figure 11:**
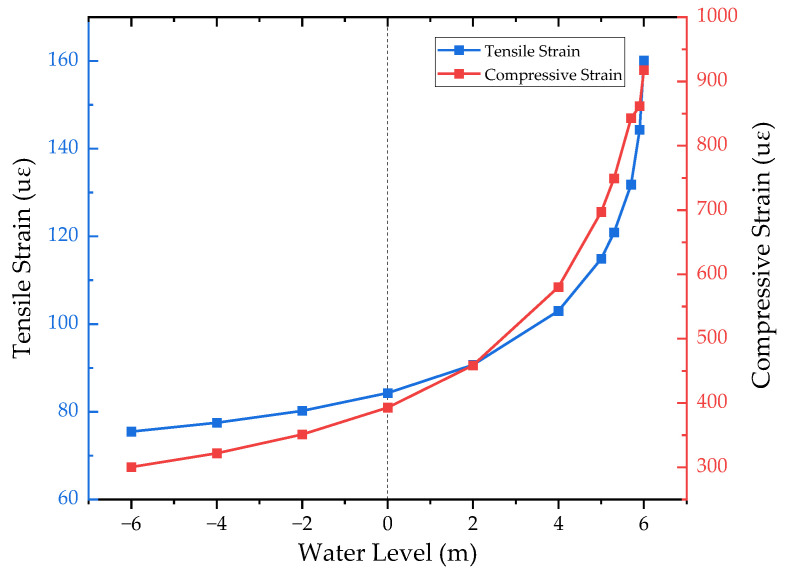
Curves of tensile and compressive strains versus water level.

From Figure 11, it can be seen that the tensile strain is similar to the exponential change with the elevation of the water level. When the water level is located underground (i.e., the water level is 0 m or less), the amount of change is slight, the water level rises from −6 m to 0 m, and the tensile strain increases 11.6%; when the water level is located above the ground, a saturated zone appears in the part of the subgrade, the tensile strain change is obvious, the water level rises from 0 m to 5 m, and the tensile strain increases by 36.3%. In particular, when the water level rises above 5 m, the tensile strain increases rapidly, corresponding to 39.3% between 5 m and 6 m.

The change rule of compressive strain was similar to that of tensile strain, increasing by 30.8%, 77.4%, and 31.6%, respectively, when the water level was at −6~0, 0~5, and 5~6 m. It can be seen that the variation in compressive strain is more obvious than that for tensile strain at a low water level, while the tensile strain better reflects the influence of the submerged water level at a high water level on the subgrade.

**Figure 12:**
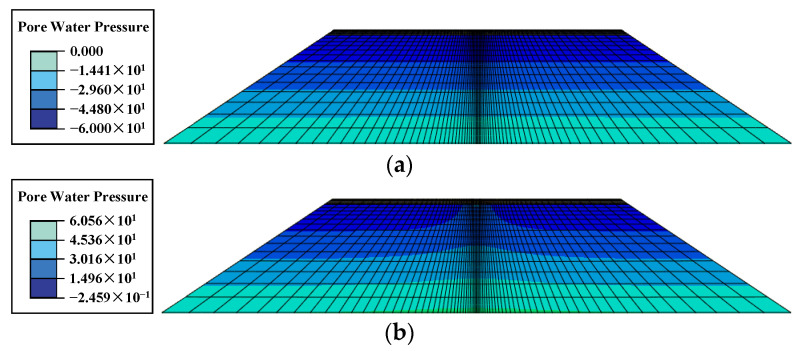
Pore water pressure distributions corresponding to peak load (**a**) at a water level of 0 m and (**b**) at a water level of 6 m.

Specifically, the rapid increase in structural strain in the high-water-level case may be due to the influence of excess pore water pressure. The pore water pressure distributions corresponding to the peak loads at water levels of 0 m and 6 m, respectively, are given in Figure 12. Since the loading time was quite short, the FWD load was loaded to the peak value in only 0.015 s, and the permeability coefficient of the soil was quite small. Therefore, the excess pore water pressure accumulated within the subgrade could not be dissipated within such a short period of time, and the effective stresses were further narrowed down, leading to a decrease in the resilient modulus of the soil. However, when the water level of the subgrade was not high, the saturated zone of the soil was located at a deeper position, and only a small amount of excess pore water pressure was induced by the transfer of the pavement load to the saturated zone; additionally, the modulus of the soil at this depth contributes a small amount to the values of the strains, and thus the change in strain is not obvious.

### 3.4. Equivalent Resilient Modulus

**Figure 13:**
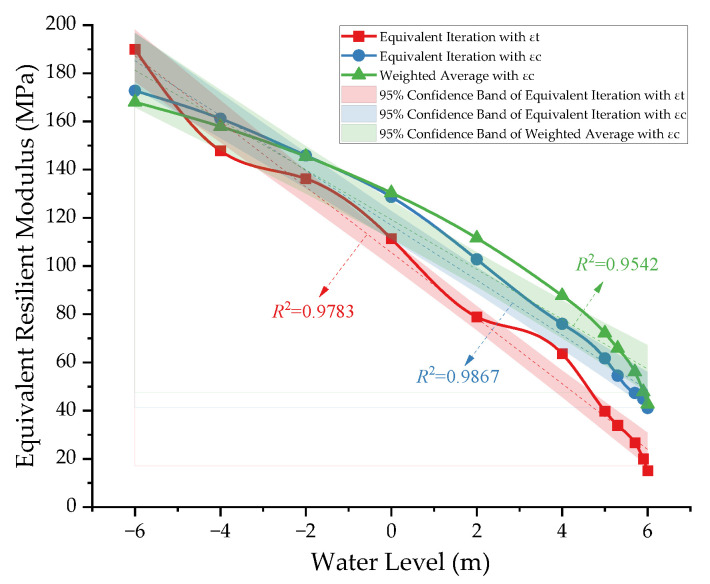
Equivalent resilient modulus results of subgrade obtained using different methods.

The two methods described in Section 2.3 were employed to calculate the equivalent resilient modulus of the subgrade, and the results are shown in Figure 13. Comparing the results of equivalent iteration by the two indicators of tensile strain *ε_t_* and compressive strain *ε_c_*, it can be seen that the modulus values of the two curves are different at the same water level due to the different indicators chosen, but the two show similar trends. The moduli of the subgrade all decrease significantly with the increase in water level. The variation range is 20~190 MPa, and the linear correlation coefficients are 0.9867 and 0.9783, respectively, which can essentially be regarded as corresponding to a linear variation.

Based on the same indicator of compressive strain *ε_c_*, the results of equivalent iteration and weighted average were compared. It was revealed that the weighted average results are in high agreement with the more accurate equivalent iteration results, so the method of determining the weighted average according to the distribution of the compressive strain can be considered feasible.

In conclusion, both methods for determining the equivalent resilient modulus value of a subgrade show a strong moisture correlation. This also implies that the effect of subgrade moisture should be emphasized in the design of actual pavement structures that and necessary anti-drainage measures should be taken. Between the two methods, the equivalent iteration method is more complicated, but its calculation results are more accurate with the aid of UMAT subroutine. The weighted average method is simpler, but the determination of the weight function is the main difficulty in this regard. The calculation method can be selected according to actual needs.

## 4. Discussion

In this section, the proposed equivalent modulus calculation method is used to analyze the influence of key influencing factors, including subgrade material and structural parameters, on the value of the equivalent resilient modulus so as to provide a reference for the design of submerged subgrades. Moreover, the suggested values for the resilient modulus of subgrade can also be presented in relation to local construction conditions.

In order to effectively control the variables, if not specified, the following simulation parameters were set so as to be consistent with those of the previous model, the water level was set to 2 m, and the compressive strain equivalent iteration method was applied to calculate the equivalent modulus. In particular, only the analysis results under several common working conditions are provided, and the reasonableness of the trend was analyzed with respect to the mechanism, while the specific values under other special working conditions need to be further explored.

### 4.1. Influence of Material Parameters

**Figure 14:**
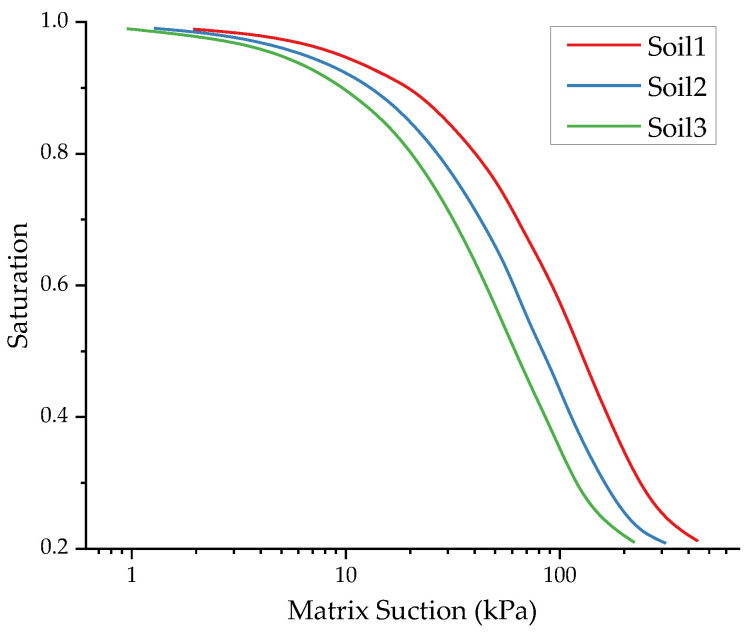
Three types of SWCC of clay.

The SWCC of unsaturated soil determines the relationship between matrix suction and saturation, constituting an important parameter in the fluid–solid coupling analysis of unsaturated soil. Meanwhile, the saturated permeability coefficient of the subgrade directly affects the speed of moisture change inside the subgrade. Therefore, three SWCCs (Figure 14) and different saturated permeability coefficients of clay subgrade were selected to investigate the influence of the above two hydraulic parameters on the results of the equivalent modulus calculation.

**Figure 15:**
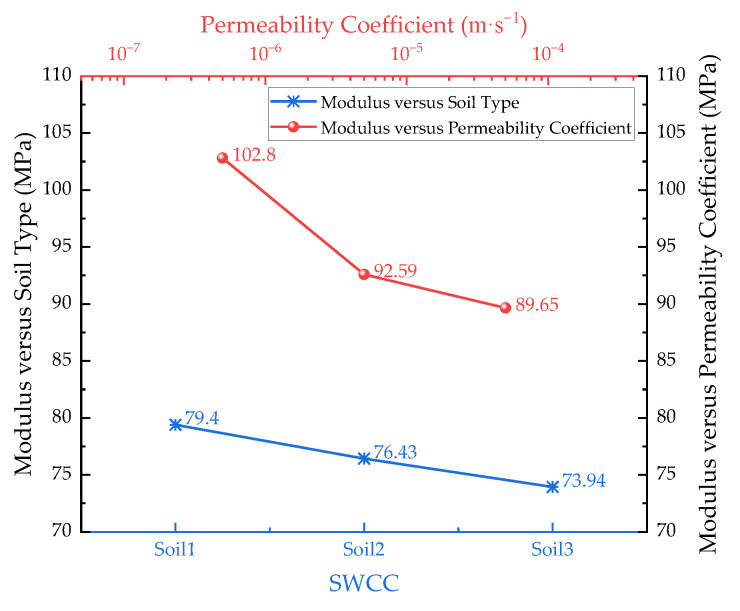
Equivalent modulus of subgrade with different hydraulic parameters.

As the SWCC gradually shifts towards the low-suction region, the saturation of the soil decreases under the same suction conditions, resulting in a decrease in the effective stress and modulus in the unsaturated zone, as shown in Figure 15. However, the magnitude of modulus reduction is not obvious, presumably due to the small permeability coefficient setting. With the continuous increase in the permeability coefficient, the modulus shows obvious attenuation. When the permeability coefficient is increased 10-fold, the modulus decreases by 9.9%, and when it is further increased 100-fold, the modulus only decreases by 3.2%, and there is a certain saturation effect, indicating that at this time, the permeability coefficient is already large enough, and the moisture at the side slope can enter the roadbed rapidly.

### 4.2. Influence of Structural Parameters

In order to investigate the influence of subgrade structural parameters on the equivalent resilient modulus, the width, height, and slope of the subgrade were selected as the key influencing factors. For the width, three conditions, namely, 12 m, 16 m, and 26 m, were selected according to different highway grades. For the height, 6, 8, and 10 m were selected. For the slope, three conditions, namely, 1:1, 1:1.5, and 1:2, were selected for analysis.

**Figure 16:**
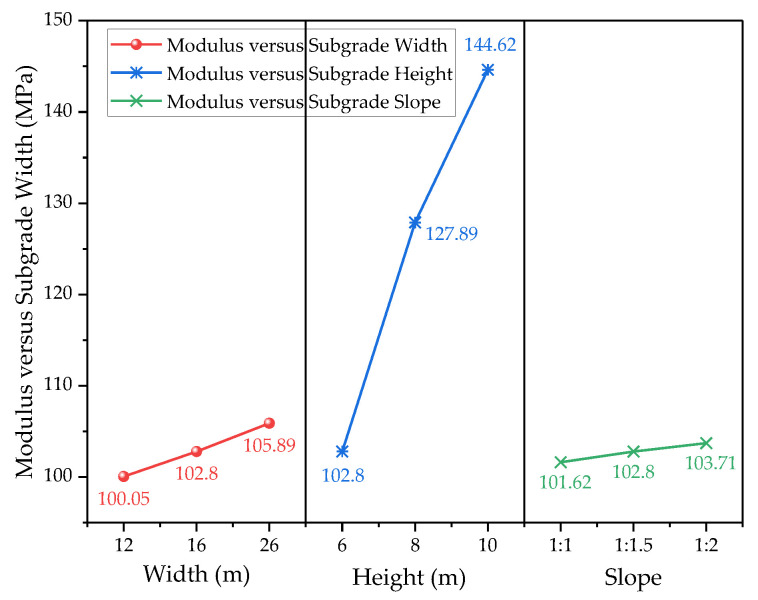
Equivalent modulus of subgrade with different structural parameters.

As can be seen in Figure 16, the equivalent modulus of the subgrade increases gradually with the increase in subgrade width, but the overall change is not large, and the modulus only increases by 5.8% when the subgrade width increases from 12 m to 26 m. The increase in subgrade width prolongs the duration of water inflow from the slope to the inside of the subgrade when the water level rises; additionally, the hysteresis phenomenon of moisture inside the subgrade is obvious, and the equivalent modulus of the subgrade increases. Conversely, if the water level decreases, the moisture inside the subgrade is discharged slowly, resulting in a larger modulus in a wider subgrade. Therefore, in the determination of the correlation between subgrade width and equivalent modulus, one should also consider the water level change conditions.

Compared with subgrade width, the effect of subgrade height on the equivalent modulus of the subgrade is fairly obvious, and the modulus increases by 40.7% when the subgrade height increases from 6 m to 8 m. With the increase in subgrade height, the range of the unsaturated zone of the subgrade enlarges, and the overall matrix suction in the unsaturated zone increases; meanwhile, the effective stress in the saturated zone of the subgrade obviously increases due to the increase in soil self-gravitational stress, which, in turn, leads to an increase in the overall modulus of the subgrade.

There are no significant differences in the results of the resilient modulus of the subgrade under different slopes. Theoretically, the effect produced by the increase in slope is similar to the increase in subgrade width; i.e., it can be regarded as a narrow subgrade when the slope rate is 1:1 and a wide subgrade when the slope rate is 1:2. However, the hysteresis effect of moisture when the slope decreases is not obvious, and therefore the effect of slope on the resilient modulus of the subgrade can nearly be neglected in the actual engineering process.

### 4.3. Design Framework for Submerged Subgrade

**Figure 17:**
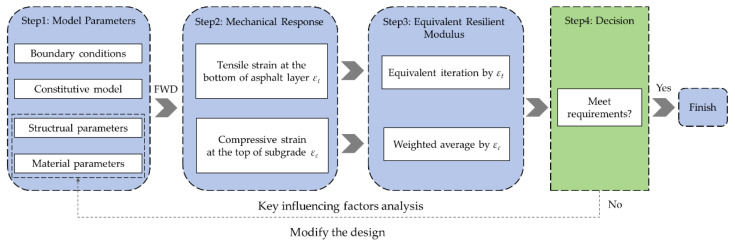
Flowchart for design of submerged subgrade.

Based on the calculation method and analysis process proposed in this study, a preliminary framework for the design of a submerged subgrade was developed, as shown in Figure 17. The framework allows designers to quickly calculate the equivalent resilient modulus of a submerged subgrade, assess whether the overall stiffness of the subgrade meets the design requirements, and guide the optimization of the initial design based on the results of the analysis of the key influencing factors. Unlike the current resilient modulus prediction model for soil samples under specified conditions, this method considers the spatial inhomogeneous distribution of the modulus of the subgrade and calculates the equivalent resilient modulus of the subgrade as a whole by means of the finite element method, which is of strong engineering practicability, and the framework can generally be divided into four parts:

1. Determine the model parameters, including boundary conditions, the constitutive model, structural parameters, and material parameters. The constitutive model of Equation (5) must be adopted, and other parameters need to be preliminarily determined in combination with local construction conditions and in consideration of engineering experience.
2. Calculate the mechanical response. Calculate the tensile strain at the bottom of the asphalt layer, *ε_t_*, and the compressive strain at the top of the subgrade, *ε_c_*, under FWD loading as representative dynamic response indicators. At the same time, these two indicators will also be used as the basis for calculating the equivalent resilient modulus of the subgrade.
3. Calculate the equivalent resilient modulus. The equivalent resilient modulus can be calculated via equivalent iteration with tensile strain and weighted average with compressive strain. Theoretically, the equivalent iteration method has higher accuracy, while the weighted average method has higher computational efficiency. Designers can choose a method according to the requirements of the task at hand.
4. Carry out decision making. Judge whether the results of the equivalent resilient modulus meet the requirements. If so, then complete this design. If not, the design parameters can be modified with reference to the results of the key influencing factors analysis given in this study until the design requirements are met.

## 5. Conclusions

In this study, a finite element model of a typical submerged subgrade was established, and a constitutive model considering the effect of saturation and matrix suction was introduced via a UMAT subroutine. The equivalent iteration and weighted average methods were used to calculate the equivalent resilient modulus. Based on this, the dynamic response and equivalent resilient modulus of the subgrade under different water levels were obtained. Finally, the influence of material parameters and the structural parameters of the subgrade were analyzed, and the results can provide a reference for the design and a suitable modulus value of the subgrade. The conclusions are as follows:

1. The effect of water level rise on the tensile strain at the bottom of the asphalt layer and the compressive strain at the top of the subgrade is obvious, and its trend is similar to an exponential change. At a low water level, the change in compressive strain is more obvious, while the change in tensile strain is more significant when the water level rises to a point where the subgrade is close to saturation. In fact, a situation in which near-saturation occurs is very rare, so the indicator of compressive strain is especially important in the design of a submerged subgrade.
2. The equivalent resilient modulus of the subgrade calculated using the equivalent iteration and weighted average methods has a strong correlation with the moisture content of the subgrade, and the modulus of the subgrade basically decreases linearly with the increase in the water level. The results of the weighted average based on the distribution of compressive strains at the top of the subgrade under FWD load are in high agreement with the results of the equivalent iteration, which is a more accurate method in theory. Therefore, it can be concluded that the method of determining the weighted average based on the distribution of compressive strain is feasible.
3. Among the subgrade materials and structural parameters considered in this study, the saturated permeability coefficient and subgrade height have the most significant effect on the resilient modulus of the subgrade, while SWCC and subgrade width have a slight effect on the modulus, and the effect of slope can be approximately ignored. Therefore, during the process of designing a submerged subgrade, the influence of the above parameters on the dynamic response of the structure should be emphasized, and the corresponding suggested values of the resilient modulus of the subgrade should be proposed according to the actual construction conditions.

This study provides an effective means for calculating and analyzing the equivalent resilient modulus of a submerged subgrade, but there is still room for improvement in this methodology. Only the results obtained under individual working conditions were considered. In fact, subgrades experience seasonal wet and dry cyclic effects, and the applicability of the constitutive model proposed in this study under such effects needs to be further discussed.

In addition, a preliminary framework for the design of a submerged subgrade was proposed in this study, but the overall process is still slightly cumbersome, and the computational efficiency is low in the case of a large quantity of data, making it to apply this framework on a wide scale in engineering practice. Therefore, it is possible to try to program the framework and develop a corresponding graphical user interface (GUI) so as to achieve rapid modelling and automatic computation, constituting the focus of our future research.

## Figures and Tables

**Table 1:** Pavement material parameters.

Layer	Material	Thickness (cm)	Elastic Modulus (MPa)	Poisson’s Ratio	Dry Density (g·cm^−3^)
Top layer	AC13	4	8200	0.25	2.40
Bottom layer	AC20	6	8000	0.25	2.40
Base course	Graded Aggregate	20	300	0.35	2.32

**Table 2:** Fitting parameters of different soil samples.

Soil Samples	Fitting Parameters			
	*k*_1_	*k*_2_	*k*_3_	*R* ^2^
Qian et al. Shanghai [24]	0.3712	0.9822	−1.6248	0.9627
Qian et al. Shandong [24]	0.3942	0.9973	−1.5841	0.9415
Liang et al. A-4 [23]	0.4544	0.8842	−1.7612	0.9638
Liang et al. A-6 [23]	0.4832	0.9161	−1.8033	0.9725

## Data Availability

The data used to support the findings of this study are available from the first author upon request. In this paper, some models and code used during the study are proprietary or confidential in nature and may only be provided with restrictions, such as the UMAT subroutine and python script in ABAQUS.
